# Clarifying the definition and handling of the early post-ablation period in studies of persistent atrial fibrillation

**DOI:** 10.1093/europace/euaf305

**Published:** 2025-11-25

**Authors:** Laurent Fauchier, Yassine Lemrini

**Affiliations:** Service de Cardiologie et Laboratoire D’Electrophysiologie Cardiaque, Centre Hospitalier Universitaire Trousseau, Université François Rabelais, Tours 37044, France; Service de Cardiologie et Laboratoire D’Electrophysiologie Cardiaque, Centre Hospitalier Universitaire Trousseau, Université François Rabelais, Tours 37044, France

We read with great interest the work by Qin et al. comparing extra-pulmonary vein extensive ablation vs. repeat in-situ ablation for recurrent persistent atrial fibrillation.^1^ The study adds important evidence on repeat ablation strategies in this challenging population. However, we noted a methodological issue that may affect the interpretation of the Kaplan–Meier (KM) curves and reported freedom-from-recurrence rates. In Figure 3, the event-free survival curves do not start at 100%, suggesting that early recurrences during the so-called ‘blanking’ period were included as events rather than being censored at baseline. According to the 2024 EHRA/HRS/APHRS/LAHRS consensus on AF ablation,^2^ the early post-ablation observation period should last eight weeks, not three months as stated by the authors. Events within this time frame are considered transient and should be excluded from endpoint analyses to prevent misclassification of temporary rhythm disturbances linked to atrial inflammation or autonomic imbalance. Recent data from continuous rhythm monitoring identified an optimal blanking duration of about six weeks, as arrhythmias beyond this point predicted late recurrence, supporting the current 8-week consensus as more physiologically relevant than the 3-month definition.^3^

Practically, a consistent approach may be defined as follows:

- Paroxysmal AF recurrence < 8 weeks: omit these episodes and define the first qualifying recurrence as that occurring > 8 weeks after ablation.
- Early persistent AF reverting to sinus rhythm before 8 weeks: omit the episode; the first qualifying recurrence is that > 8 weeks.
- Persistent AF ongoing at 8 weeks: either exclude the patient from recurrence analysis or initiate follow-up at the time sinus rhythm is restored (typically after cardioversion > 8 weeks).

Adhering to this framework ensures that survival curves begin at 100% and that the time origin corresponds to the start of valid follow-up—i.e. once the post-ablation healing phase has ended. Inclusion of early events or patients continuously in AF during this period artificially lowers initial survival estimates and may exaggerate between-group differences. Accurate application of the early post-ablation (or ‘blanking’) definition is crucial for comparability across studies and registries, as emphasized by the EuroHeart/EHRA methodological standards.^4^ Transparent reporting of event definitions and time-to-follow-up initiation would therefore enhance interpretability and alignment with international consensus. We commend the authors for addressing a key clinical question and hope these clarifications will contribute to more standardized analyses of ablation outcomes in persistent AF.
